# Treatment of early-stage HER2+ breast cancer—an evolving field

**DOI:** 10.3332/ecancer.2015.523

**Published:** 2015-04-16

**Authors:** Arlindo R Ferreira, Kamal S Saini, Otto Metzger-Filho

**Affiliations:** 1 Dana-Farber Cancer Institute, 450 Brookline Ave, Boston MA 02215, USA; 2 Hospital de Santa Maria and Instituto de Medicina Molecular, Universidade de Lisboa, Av. Prof. Egas Moniz, 1649-035—Lisbon, Portugal; 3 Institut Jules Bordet, Université Libre de Bruxelles, Boulevard de Waterloo 121, 1000, Brussels, Belgium

**Keywords:** breast neoplasm, trastuzumab, neoadjuvant therapy, adjuvant therapy, antibodies, monoclonal, humanised

## Abstract

The evolving field of HER2-targeted therapy has significantly improved the outcome of women diagnosed with HER2-positive invasive breast cancer. In this review, we sought to summarise the efficacy of trastuzumab-based regimens in the adjuvant and neoadjuvant setting with a special emphasis on relevant clinical questions: treatment duration, sequence of trastuzumab administration, toxicity, the role of anthracycline-based regimens, and optimal management of small HER2+ tumours. Controversial topics are discussed taking into consideration the development of modern anti-HER2 agents.

## Background

Breast cancer (BC) is a heterogeneous disease with several biological subtypes. Human epidermal growth factor receptor 2 (HER2) is a strong phenotype determinant present in approximately 20% [1] of all BCs. Directed therapy towards this receptor with trastuzumab, a humanised monoclonal antibody, decisively contributed to a prognostic improvement. This was first demonstrated in the metastatic setting and subsequently in early-stage BC. In this article, the authors review the role of trastuzumab in early-stage BC.

## Trastuzumab: clinical efficacy in the adjuvant setting

In the adjuvant setting, anti-HER2-targeted therapy with trastuzumab greatly contributed to improvement of clinical outcomes, as measured by disease-free survival (DFS) and overall survival (OS). Several multicentre international trials were designed to assess the role of trastuzumab treatment in high-risk early-stage BC patients, defined as node positive or node negative with tumours larger than 1 cm [2–5] or 2 cm [6, 7] (largely patients with stage II or stage III BC). The key features of these trials are summarised in Table 1.

These findings were further scrutinised in a meta-analysis that gathered the results of eight trials (total 11991 patients) testing the benefit of trastuzumab when added to adjuvant chemotherapy versus chemotherapy alone [8]. With a median follow-up of three years, trastuzumabcontaining treatments significantly improved OS (hazard ratio [HR] 0.66, 95% confidence interval (CI) 0.57–0.77, and disease-free survival (DFS) (HR 0.60, 95% CI 0.5–0.71). Despite efficacy improvement, cardiac toxicity was documented in a minority of cases, with trastuzumabtreated patients being more likely to suffer from congestive heart failure (CHF; relative risk [RR] 5.11, 90% CI 3–8.72) and left ventricular ejection fraction (LVEF) decline (RR 1.83, 90% CI 1.36–2.47). Currently, trastuzumab is the only anti-HER2 drug that has demonstrated a survival benefit in the adjuvant setting.

Recently, trastuzumab was also prospectively tested in a cohort of patients mainly with stage I HER2-positive BC [9]. The anticipated small absolute benefit and the expected adverse events led to the exclusion of most of these patients in the pivotal studies of trastuzumab. In this phase II, uncontrolled, single-arm and multicentre study, 406 patients with tumours with less than 3 cm and mostly node negative disease (1.5% patients had N1mic) were treated with adjuvant paclitaxel (80 mg weekly) plus trastuzumab (4 mg/kg loading dose followed for 2 mg/kg weekly) for 12 weeks, followed by trastuzumab (either 2mg/kg weekly or 6 mg/kg every three weeks) for a total of 40 weeks, thus omitting any anthracycline or platinum agent. With a median follow-up of four years, the investigators reported an invasive disease-free survival at three years of 98.7% (95% CI 97.6–99.8). Among the patients with disease recurrence, two (0.4%) had distant events. A 6% withdrawal rate because of adverse events was also reported. The most relevant adverse events included grade 3 neuropathy (3.2%; 95% CI 1.7–5.4), symptomatic but reversible congestive heart failure (0.5%; 95% CI 0.1–1.8) and asymptomatic declines in ejection fraction (3.2%; 95% CI 1.7–5.4). Based on the comparison to historical data, the authors argue that the risk of cancer recurrence and serious toxic events were low.

Finally, population-based studies reported reassuring patient safety outcomes and treatment compliance [10–13].

## Trastuzumab: clinical efficacy in the neoadjuvant setting

Neoadjuvant therapy is the standard approach for treating locally advanced and inflammatory BCs; however, it can be also considered an equally valid strategy for the treatment of early-stage BCs [14]. In HER2-positive BCs, a pathologic complete response (pCR) rate of around 31–50% (for hormone receptor-positive and negative respectively) is expected [15]. In these cases, pCR is a prognostic marker of favourable long-term outcomes, as measured by event free survival (EFS; HR 0.39; 95% CI 0.31–0.50) and OS (HR 0.34; 95% CI 0.24–0.47) [15].

In the neoadjuvant setting, trastuzumab has been tested in combination with chemotherapy and other anti-HER2 agents (Table 2). The ‘first generation’ trials addressed the efficacy of trastuzumab plus chemotherapy when compared to chemotherapy alone. The MD Anderson study [16] was a single centre, phase III trial that randomly assigned 42 of 164 planned patients with stage II–IIIA invasive but non-inflammatory BC to either paclitaxel followed by fluorouracil, epirubicin, and cyclophosphamide (FEC) with or without trastuzumab. The primary endpoint was pCR rate. The data and safety monitoring board stopped this study early given the significant differences in clinical response between arms favouring the use of trastuzumab: pCR rate of 65.2% (95% CI, 43–84%) versus 26.3% (95% CI, 9–51%), p = 0.016. At a median follow-up of three years, no DFS events were recorded in the trastuzumab arm, while in the arm without trastuzumab 94.7% (95% CI, 85.2–100%) were DFS-free at one year and 85.3% (95% CI, 67.6–100%) were DFS-free at three years (p = 0.041) (17). No patients receiving chemotherapy plus trastuzumab developed clinical cardiac dysfunction or cardiac related deaths. The NOAH study [18] provided further insight into the efficacy of trastuzumab. This multicentre, open-label, phase III trial randomly assigned 235 patients with locally advanced or inflammatory BC to either neoadjuvant trastuzumab plus a chemotherapy regimen based on doxorubicin, paclitaxel, cyclophosphamide, methotrexate, and fluorouracil followed by adjuvant trastuzumab, or neoadjuvant chemotherapy only. It is noteworthy that patients allocated to chemotherapy alone later received one year of adjuvant trastuzumab after the final positive results of the trastuzumab arm. With a median follow-up of three years, the neoadjuvant trastuzumab arm demonstrated a 41% event-free survival (EFS) improvement (HR 0.59, 95% CI 0.38–0.90; p = 0.013) for primary endpoint. The pCR rate was also higher in the trastuzumab arm (38% versus 19%, p = 0.001). Long term results with a median follow-up of 5.4 years confirmed the previous EFS results (46% improvement; 95% CI 7–56%) and reported an 41% improvement in five-years BC specific survival (BCSS; HR 0.59, p = 0.023) [19]. Finally, the GeparQuattro [20] was a prospective study that enrolled 1509 patients with locally advanced BC, from which 451 HER2-positive and 1058 HER2-negative, to receive epirubicin and cyclophosphamide (EC) and then be randomly assigned to either docetaxel, docetaxel plus capecitabine, or docetaxel followed by capecitabine. Those patients with HER2-positive BC further received concomitant trastuzumab for a total period of one year. A pCR (primary endpoint) rate of 31.7% was observed in those patients receiving trastuzumab (with HER2-positive disease). In comparison, only 15.7% of the patients achieved a pCR in the reference group of HER2-negative patients. These trials supported the current use of trastuzumab in association with chemotherapy in the neoadjuvant setting.

Subsequently, the ‘second generation’ trials compared trastuzumab to other anti-HER2 agents or the added benefit of other anti-HER2 agents to trastuzumab plus chemotherapy. The GeparQuinto trial [21] performed a head-to-head comparison between trastuzumab and lapatinib. This was a multicentre, open-label, phase III trial that randomly assigned 620 patients with locally advanced BC to epirubicin plus cyclophosphamide and docetaxel with either trastuzumab or lapatinib. The primary endpoint was the pCR rate, which was of 30.3% in the trastuzumab arm, and of 22.7% in the lapatinib arm (OR 0.68, 95% CI 0.47–0.97; p = 0.04) revealing a higher activity of trastuzumab. Lapatinib was also tested in association with trastuzumab. The CHER-LOB study [22] was a phase II trial that randomised 121 stage II to IIIA operable BC patients to trastuzumab, lapatinib, or both. The primary endpoint was the rate of pCR, which was superior in the combination arm (46.7%, 90% CI 34.4–58.9%). The trastuzumab only arm had a pCR of 25% (90% CI 13.1–36.9%) and the lapatinib only arm 23.6% (90% 14.5–38.1%). The association lapatinib-trastuzumab was further tested in the NeoALTTO study [23], a multicentre, open-label, phase III trial that randomly assigned 455 patients with tumours greater than 2 cm to either lapatinib, trastuzumab, or both. The primary endpoint was pCR rate, which was higher in the group receiving combination therapy (51.3%, 95% CI 43.1–59.5; versus 29·5%, 95% CI 22.4–37.5 in the trastuzumab arm; p = 0·0001 for the difference). No significant difference was documented between the trastuzumab and lapatinib arms (lapatinib pCR of 24.7%, 95% CI 18.1–32.3; p = 0.34 for the difference). The authors concluded that the dual inhibition with trastuzumab and lapatinib might be a valid option in the neoadjuvant setting. Of note, less impressive findings were subsequently documented in the ALTTO study that tested the adjuvant use of the same combination of trastuzumab plus lapatinib in the adjuvant setting (discussed ahead) [24]. Other studies testing this combination are summarised in Table 2.

Besides lapatinib, trastuzumab was also tested in combination with pertuzumab. The NeoSphere trial [25] was a multicentre, open-label, phase II trial that randomly assigned 417 patients with early, locally advanced, or inflammatory BC to four therapeutic groups: trastuzumab plus docetaxel (group A), pertuzumab and trastuzumab plus docetaxel (group B), pertuzumab and trastuzumab (group C), and pertuzumab plus docetaxel (group D). After surgery, patients who received docetaxel, further received FEC, and then completed trastuzumab, while the group C received docetaxel, FEC, and afterwards completed one year of trastuzumab. The primary endpoint was pCR (in the breast). The trial revealed that patients treated with docetaxel, trastuzumab, and pertuzumab (group B) had a more favourable pCR rate (45.8%, 95% CI 36.1–55.7) when compared with those treated with docetaxel plus trastuzumab (group A, 29.0%, 95% CI 20.6–38.5; p = 0·014 for the difference). Groups C and D had a pCR of 16.8% (95% CI 10.3–25.3) and 24.0% (15.8–33.7), respectively. In this study, the combination of trastuzumab to pertuzumab did not increase cardiac toxicity. Besides showing that the combination of two anti-HER2 agents (trastuzumab and pertuzumab) improves the rates of pCR, it was also noteworthy that an arm with dual HER2 blockage (trastuzumab + pertuzumab; group C) is *per se* effective in achieving pCR in a subset of patients without any additional chemotherapy.

## Optimal duration of therapy

Only recently was the treatment duration of trastuzumab more clearly established. The pivotal trials studying the role of trastuzumab in early BC used an empirical reference therapy duration of one year [2–4]. However, the potential for improved efficacy versus the need for reduced toxicity led to an exploration of different treatment durations.

The FinHER trial [6] tested a shorter duration of adjuvant trastuzumab in a subset of early BC patients with HER2-positive high-risk cancers (axillary-node-positive or node-negative with breast tumour mass ≥2 cm). In this multicentre, open-label, phase III trial, 1010 patients were randomly assigned to adjuvant docetaxel or vinorelbine followed by FEC with or without nine cycles of weekly trastuzumab in the subset of HER2-positive breast cancer (232 patients, 22.97%). The primary endpoint was recurrence-free survival (RFS). At a median follow-up of 37 months, trastuzumab was an effective therapy (RFS HR 0.41, 95% CI 0.21–0.83, p = 0.01; OS HR 0.41, 95% CI 0.16–1.08, p = 0.07). None of the women exposed to trastuzumab had heart failure. The documented clinical improvement with the addition of trastuzumab was similar to those trials with standard one-year therapy [2, 3], and with a potential improvement in the toxicity profile.

Moreover, the phase III study PHARE [26] randomly assigned 1693 HER2-positive high-risk patients (axillary-disease positive) that had already received at least four cycles of chemotherapy and six months of trastuzumab to continue trastuzumab for an additional period of six months or to discontinue trastuzumab. This was a non-inferiority trial with DFS as primary outcome. At a median follow-up of three-and-onehalf years, six months of trastuzumab could not demonstrate non-inferiority (DFS HR 1.28, 95% CI 1.05–1.56, p for non-inferiority = 0.29) (Table 3). Furthermore, fewer patients in the 12-month arm had distant recurrences as first DFS event (108 versus 141 events; HR 1.33, 95% CI 1.04–1.71). These unfavourable results were also observed for OS (66 versus 93 events; HR 1.46, 95% CI 1.06–2.01), despite the need for additional accumulation of events. The trial had preplanned sub-group analyses by hormone-receptor status and timing of administration of chemotherapy and trastuzumab (sequential or concomitant). The ER-negative cancers treated with sequential chemotherapy and trastuzumab had the worst two year DFS (89.8%, 95% CI 85.8–92.7 versus 84.5%, 95% CI 80.0–88.1), which the authors argue contributed more decisively to the unfavourable results of six months of trastuzumab. Concerning safety, similar rates of serious adverse events were documented; however early stopping of trastuzumab because of cardiac toxicity was more common in the 12-month arm (103 events or 6.1% versus 32 events or 1.9%). Finally, other studies under development are further testing shorter treatment regimens of adjuvant trastuzumab. Comparing 6 to 12 months: the Hellenic group trial (NCT00615602; enrolled 489 patients) and the Persephone study (NCT00712140; plans to enrol 4000 patients); while comparing 3 to 12 months: the SOLD study (NCT00593697; enrolled 2168 patients) and Short-Her study (NCT00629278; plans to enrol 2500 patients).

In search of improved efficacy, the HERA trial, an international, multicentre, open-label phase III trial, besides comparing one year of adjuvant trastuzumab with observation after standard adjuvant chemotherapy, also enrolled patients to a third arm treated with two years of trastuzumab [27]. This trial was designed to detect DFS superiority of the two-year treatment arm and included patients without evidence of disease at 12 months after randomisation (landmark analysis). At a median follow-up of eight years, the results showed no significant difference between groups in terms of DFS (HR 0.99, 95% CI 0.85–1.14, p = 0.86) or OS (HR 1.05, 95% CI 0.86–1.28, p = 0.63) (Table 3). The results were further explored in preplanned secondary analysis by hormone receptor status, without any difference between subgroups. Interestingly, the hormone-receptor-negative subgroup receiving two years of trastuzumab had a transient but not statistically significant improvement in DFS. The authors propose that this may represent a short-term augmented risk of relapse during a period when no adjuvant therapy is given to these patients (no hormone, nor trastuzumab therapy). The authors argue that this finding is specially supported by the fact that the cohort of hormone-receptor-negative patients receiving a second year of trastuzumab closely resembles that of the hormone-receptor-positive groups. Regarding safety, severe symptomatic cardiac endpoints (CHF NYHA III-IV with a LVEF decline ≥10% from baseline and to an absolute LVEF <50%, or cardiac death) were similar between both arms (14 events or 0.8% versus 16 events or 1%). On the other hand, asymptomatic or mildly symptomatic cardiac endpoints (CHF NYHA I-II with a LVEF decline ≥10% from baseline and to an absolute LVEF <50%) were more common in the two-year arm (69 events or 4.1% versus 120 events or 7.2%). Based on these results the authors concluded that two years of adjuvant trastuzumab has an unfavourable risk-benefit ratio.

Grounded in the current evidence, one year of adjuvant trastuzumab remains the standard treatment duration.

## Anthracycline use and treatment sequence

Anthracycline-based chemotherapy regimens were used in the majority of the trials evaluating trastuzumab in the adjuvant setting (Table 1). As previously discussed, cardiotoxicity, manifested as CHF or asymptomatic LVEF decline, was identified as a source of concern with adjuvant trastuzumab therapy, which is aggravated with the use of anthracycline-based chemotherapy regimens [28–30]. With the rationale of enhancing cardiac safety of HER2-positive BC adjuvant therapy, the BCIRG-006 study [4] tested the efficacy of an anthracycline-free chemotherapy regimen. This international, multicentre, open-label, phase III trial randomised 3222 patients with HER2-positive high-risk T1–T3 breast cancer to receive a standard adjuvant anthracycline-taxane containing chemotherapy regimen (AC-T), the same chemotherapy regimen plus trastuzumab (AC-TH), or an anthracycline-free chemotherapy regimen containing docetaxel, carboplatin, and trastuzumab (TCH). In this trial, high risk was defined as node positive or node negative with high-risk features (hormone receptor negative, histologic grade 2–3, tumour >2 cm, and age at diagnosis <35 years). The primary endpoint was DFS. With a median follow-up of approximately five years, AC-T, AC-TH, and TCH arms had an estimated DFS of 75%, 84%, and 81%, respectively; the HR for DFS comparing AC-T to AC-TH and AC-T to TCH were 0.64 and 0.63, respectively (both p < 0.001). The AC-T, AC-TH, and TCH arms had an OS of 87%, 92%, and 91%, respectively; the HR for OS comparing AC-T to AC-TH and AC-T to TCH were 0.63 (p < 0.001) and 0.77 (p = 0.04), respectively. The numerical differences in OS and DFS between AC-TH and TCH were statistically non-significant. Concerning safety, the occurrence of CHF was more common in the group exposed to AC-TH (2.0%) than in AC-T (0.7%) or TCH (0.4%). This difference was also evident for asymptomatic decline in LVEF (>10% drop). The group receiving AC-TH had the highest incidence of LVEF decline (18.6%) when compared to AC-T (11.2%) and TCH (9.4%). Differences in CHF and LVEF decline between in AC-TH and TCH groups were statistically different (p < 0.001). Neutropaenia and leukopaenia were also more frequent in the AC-TH arm (without an impact on febrile neutropaenia), while thrombocytopaenia and anaemia were more frequent in the TCH arm. Sensory and motor neuropathy was more frequent in the AC-TH arm. Grounded on these results, the authors highlighted potential advantages of the TCH approach over AC-TH: similar efficacy between the two regimens (while recognising that the study was not powered for this comparison), reduced CHF and haematologic toxicity (myelodysplasia and acute leukaemia), and shorter adjuvant treatment duration (12 versus 16 weeks). This evidence was sufficient for the authors to conclude ‘the risk-benefit ratio favoured the non-anthracycline TCH regimen over AC-T plus trastuzumab’. Others [31] disagree with this conclusion, based mainly on lack of power of BCIRG-006 trial to demonstrate equivalence or non-inferiority between the two chemotherapy regimens; the absence of other prospective studies demonstrating the same conclusion; and the uncertain benefit from carboplatin when compared to anthracyclinetaxane based chemotherapy in HER2-positive BC.

Whereas some of the pivotal trials opted to give trastuzumab after chemotherapy (sequential approach), others preferred to administer trastuzumab concurrent with paclitaxel (Table 1). The NCCTG N9831 study design evaluated the sequential versus concurrent approach [32]. In this multicentre, open-label, phase III study, 2448 patients received adjuvant doxorubicin and cyclophosphamide every three weeks for four cycles, and they were then randomly assigned to receive one of three options: weekly paclitaxel for 12 weeks (arm A), weekly paclitaxel plus sequential weekly trastuzumab for 52 weeks (arm B), or weekly paclitaxel plus concurrent trastuzumab for 12 weeks followed by weekly trastuzumab for 40 weeks (arm C). The primary endpoint was DFS. After a median follow-up of six years and when comparing arm B (n = 954) to C (n = 949), those receiving concurrent trastuzumab (arm C) presented a strong trend towards a reduction in the risk of DFS (HR 0.77, 99.9% CI 0.53–1.11; p-value 0.0216; the p-value superior to the prespecified boundary of 0.00116 for interim analysis).

Finally, some studies also assessed the sequence of chemotherapies (i.e. anthracycline and taxane) given in combination with trastuzumab. A randomised phase III neoadjuvant clinical trial [33] tested anthracycline followed by taxane or the reverse order, concomitantly with trastuzumab. A total of 282 patients were randomly assigned (1:1 ratio) to four cycles of FEC followed by weekly paclitaxel plus trastuzumab or the reverse sequence, with continued trastuzumab during FEC. No significant differences in pCR in the breast (primary outcome) were noted (56.5% versus 54.2% in the reverse sequence/concurrent trastuzumab arm). Grade 3–4 neutropaenia, LVEF decline, fatigue, and neurosensory disability were more frequent in reverse sequence arm.

## Formulation differences

The studies leading to the approval of trastuzumab used an intravenous (IV) formulation of the drug. While IV trastuzumab has already been shown to be a cost-effective therapy [34], its administration still requires a considerable amount of time, as the establishment of an IV line has its implications on hospitals’ logistics, finances, and patients’ quality of life. These obstacles opened the opportunity for newer formulations. In the Hannah study [35] a newer subcutaneous (SC) formulation was explored. In this phase III non-inferiority trial that included 596 BC patients eligible for neoadjuvant therapy, an IV formulation (loading dose of 8 mg/kg over 90 minutes followed by 6 mg/kg over 30–90 minutes) was compared to a SC formulation (600 mg fixed dose administered in the thigh for over about five minutes), both every three weeks and in combination with docetaxel times four followed by FEC times four (1:1 ratio between arms). Following surgery both anti-HER2 agents were continued till the completion of one year. The primary outcome was pCR and serum trough concentration at a pre-dose cycle eight before surgery. A pCR rate of 40.7% versus 45.4% was obtained in the IV versus SC formulations (95% CI for the difference -4.0 to 13.4). Moreover, a non-inferior serum trough concentration at pre-dose cycle eight before surgery was also obtained (69.0 μg/mL versus 51.8 μg/mL in the IV group; geometric mean ratio of C (trough) 1.33, 90% CI 1.24–1.44). Hence, SC trastuzumab was considered non-inferior to the IV formulation after meeting both co-primary endpoints. However, a higher rate of patients in the SC arm had serious adverse events (SAE; 21% versus 12% in the IV arm), including febrile neutropaenia (5.7% versus 3.4% in the IV arm) and infections (8.1% versus 4.4% in the IV arm). Four of these SAEs led to death, three of which in the SC arm and of which two considered being treatment related. Results from this trial led to regulatory approval of the SC formulation by the European Medicines Agency. More recently, the PrefHer study revealed that 91% of the patients’ preferred the SC formulation [36].

## Early predictors of response to trastuzumab or other anti-HER2 therapies

HER2 overexpression remains the only validated predictive marker of response to anti-HER2 therapy, even though the response to anti-HER2 directed therapies based on this marker is not homogeneous. The development of markers that would allow selecting patients with the highest likelihood to respond would be of much benefit to maximise benefit and decrease harm.

In the neoadjuvant setting, ^18^F-FDG positron emission tomography (PET)/computed tomography (CT) imaging is being tested as a predictor of the likelihood of obtaining a pCR. The NeoALTTO trial [23] tested the predictive value of ^18^F-FDG PET/CT to identify those patients with an increased likelihood of obtaining a pCR [37]. This trial had a key design feature consisting of a six week ‘biologic window period’, during which only anti-HER2 drugs were provided. Only after this period could a chemotherapy begin (paclitaxel). During this six-week interval three ^18^F-FDG PET/CT were performed: at baseline, two and six weeks. From a sample of 86 patients, a total of 62 completed the three evaluations. After two weeks of anti-HER2 therapy metabolic response was evident in the tumour and the degree of response at two weeks correlated strongly with the metabolic response at six weeks (R^2^ 0.81). PET/CT responders had a two-fold likelihood of achieving a pCR (week two: 42% versus 21%, p = 0.12; week six: 44% versus 19%, p = 0.05). PET/CT may therefore be a useful non-invasive marker of anti-HER2 response. These results are being further explored in a subset of patients in the ongoing NeoPHOEBE study (NCT01816594), a phase II study evaluating the role of a new oral PI3K inhibitor in the neoadjuvant treatment of BC.

In the metastatic setting, HER2 molecular imaging using ^89^Zirconium-labeled trastuzumab is being tested as a noninvasive whole-body imaging technique to determine tumour HER2 expression status and the localisation of tumour lesions HER2-positive, especially those inaccessible to biopsy [38], The IJBMNZrT003 trial (NCT01420146) is a phase I trial testing this diagnostic potential of HER2-positive tumours using ^89^Zirconium-labeled trastuzumab.

## Other anti-HER2 therapies

Despite trastuzumab efficacy in the treatment of HER2-positive BC, tumour relapse and resistance to therapy is common [39]; hence, other agents targeting HER2 receptor were developed.

Lapatinib is a small molecule that operates as a dual tyrosine kinase inhibitor blocking HER2 and epidermal growth factor receptor (EGFR). Lapatinib is currently not indicated in the treatment of HER2-positive BC in the adjuvant setting either as a monotherapy or in combination with trastuzumab. The TEACH investigators addressed the role of adjuvant lapatinib as a monotherapy in a randomised, placebo-controlled, phase III, and multinational trial that assigned 3161 women to lapatinib or placebo (1:1 ratio). With a median follow-up of almost four years, only a non-statistically significant trend towards lapatinib was found in terms of DFS (HR 0.83, 95% CI 0.70–1.00, p = 0.053; primary endpoint). However, no difference in terms of OS was observed (HR 0.99, 95% CI 0.74–1.31, p = 0.96). Lapatinib was also tested in association with trastuzumab in non-metastatic BC in the ALTTO study, a randomised open-label phase III trial [24]. As defined by the study group [40], the study has three study designs: ‘In Design 1, all (neo)adjuvant chemotherapy is completed prior to administration of the study treatments. In Design 2, all anthracycline-based (neo)adjuvant chemotherapy is completed prior to administration of the study treatments, while taxane is given concurrently with the study treatments. In Design 2B, a non-anthracycline regimen containing docetaxel and carboplatin is given concurrently with study treatments’. Within each of the design options patients were randomised to receive one of the following treatments: trastuzumab alone; lapatinib alone (until August 2011); trastuzumab followed by lapatinib; or lapatinib in combination with trastuzumab. The primary endpoint was DFS. An interim analysis after a median follow-up of four-and-one-half years and 555 DFS events revealed a non-significant trend towards improved DFS in the combination arm (HR 0.84, 97.5% CI 0.70–1.02; P = 0.048; four years DFS 88% versus 86%). This result contrasted with the significant improvement in terms of pCR of the combination therapy in the NeoALTTO study [23]. In light of the ALTTO study, there is no current support for the use of lapatinib in the adjuvant setting.

Pertuzumab is a recombinant humanised monoclonal antibody that targets the extracellular subdomain II of HER2 blocking receptor dimerisation (Table 4) [41]. Its mechanism of action is considered complementary to that of trastuzumab [42]. The CLEOPATRA trial [43] compared the progression-free-survival (PFS) between the combination of pertuzumab plus trastuzumab plus docetaxel and the combination of placebo plus trastuzumab plus docetaxel in 808 patients with HER2-positive metastatic BC. In the updated results from ESMO 2014 at a median follow- up of 50 months, this randomised, phase III trial demonstrated a benefit from pertuzumab when compared to the placebo (OS HR 0.68, 95% CI 0.56–0.84, p < 0.001; 56.6 months versus 40.8 months respectively) without increased cardiac toxicity. The PERUSE (NCT01572038) and VELVET (NCT01565083) studies are currently testing pertuzumab in metastatic BC in association with other chemotherapy regimens. In the neoadjuvant setting two phase II trials, the NeoSphere [25] (previously discussed) and TRYPHAENA trials [44], showed promising results in the pertuzumab plus trastuzumab plus docetaxel association when compared to the same schema without pertuzumab. Given the positive results of pertuzumab in association with trastuzumab in the metastatic and neoadjuvant setting, this combination is being further explored in the adjuvant setting. The APHINITY trial (NCT01358877) is a phase III, multinational, multicentre, double-blind, placebo-controlled trial comparing the efficacy and safety of chemotherapy plus trastuzumab and pertuzumab with that of chemotherapy plus trastuzumab and placebo as adjuvant therapy in patients with primary HER2-positive BC. The trial has completed recruitment (n = 4805) in August 2013. The primary endpoint is invasive DFS (IDFS).

Trastuzumab emtansine (T-DM1) is a conjugate between trastuzumab antibody and emtansine (DM1), a cytotoxic drug (Table 4) [45]. In the metastatic setting, the EMILIA trial compared the combination of lapatinib plus capecitabine versus T-DM1 in 991 patients with HER2-positive BC previously treated with trastuzumab and a taxane in terms of PFS. This randomised, phase III trial demonstrated an improvement from the T-DM1 arm when compared to lapatinib plus capecitabine, not only in terms of PFS (9.6 months versus 6.4 months, respectively; HR 0.65, 95% CI 0.55–0.77, p < 0.001) but also OS (30.9 months versus 25.1 months, respectively; HR 0.68, 95% CI 0.5–0.85, p < 0.001) in patients previously treated with trastuzumab. In the adjuvant setting, the KATHERINE trial (NCT01772472, started recruiting in January 2013) compares the DFS (primary endpoint) between T-DM1 versus trastuzumab as adjuvant therapy in patients with HER2-positive BC who have residual disease in the breast or axillary lymph nodes after preoperative therapy. The estimated recruitment target is 1484 patients (1:1 ratio). Also in the adjuvant setting, the KAITLIN study (NCT01966471, started recruiting in January 2014) is comparing T-DM1 plus pertuzumab to trastuzumab plus pertuzumab plus a taxane, both followed by an anthracycline. The estimated recruitment target is 2500 patients (1:1 ratio). Of note, the comparator arm (trastuzumab plus pertuzumab plus a taxane) is not a standard of care and is being currently tested in the APHINITY trial.

## Trastuzumab plus other anti-HER2 therapies (without chemotherapy)

As previously discussed, trastuzumab significantly enhanced the clinical outcomes in patients with ‘high risk’ HER2-positive BCs, i.e. tumours larger than 1 cm [2–4] or 2 cm [6]. Historically, tumours of less than 1 cm size (T1_a-b_) were considered to be ‘low risk’ tumours and often considered not to benefit from anti-HER2 directed therapy. However, some retrospective studies [46–48] and a prospective phase II study questioned this ‘low risk’ assumption [9]. Yet, standard trastuzumab plus chemotherapy regimens have well-documented toxicities, thus making the prospect of regimens containing anti-HER2 therapies with less intense chemotherapy regimens, even if applicable only in a minority of the BC patient population [49].

The NeoSphere trial [25] (previously discussed) randomly assigned patients with early, locally advanced, and inflammatory BC to trastuzumab plus docetaxel or pertuzumab and trastuzumab plus docetaxel or pertuzumab and trastuzumab or pertuzumab plus docetaxel. The pCR rates between arms were 29%, 45.8%, 16.8%, and 24%, respectively. It was promising to note the low but significant proportion of patients receiving only trastuzumab plus pertuzumab without chemotherapy that achieved a pCR (16.8%), which raised the possibility that a small minority of patients don’t benefit from chemotherapy when receiving trastuzumab plus pertuzumab. This observation was especially valid for ER-negative patients, who achieved a pCR in 29% of the cases. Other predictive biomarkers of response could help selecting those patients that do not benefit from chemotherapy.

## Conclusion and future perspectives

HER2 blockade has brought significant clinical benefit with acceptable toxicity, leading to a paradigm shift in the management of this population with hitherto poor prognosis. The preliminary data from combining new anti-HER2 therapies with trastuzumab are exciting, and several large studies are underway to validate these in the neoadjuvant and adjuvant settings.

## Conflict of interest

The authors declare no conflicts of interest.

## Figures and Tables

**Table 1.** Summary of results from major trials addressing trastuzumab benefit in the adjuvant treatment of BC.

Study	Number patients/median follow-up	Comparison treatment	DFS (compared to chemotherapy alone)	OS (compared to chemotherapy alone)	Reference
BCIRG 006 trial (ǂ)	3222/5 years	• AC → docetaxel • AC → docetaxel + trastuzumab → trastuzumab • Docetaxel + carboplatin + trastuzumab → trastuzumab	• HR 0.64, p < 0.001; 0.75, p = 0.04 • 75% versus 84% versus 81% • 257 versus 185 versus 214 events	• HR 0.63, p < 0.001; HR 0.77, p = 0.04 • 87% versus 92% versus 91% • 489 versus 290 events	Slamon *et al* (2011) [4]
NCCTG N9831 trial (ǂ and §)	2184/6 years (DFS analysis at five years follow—up)	• AC → paclitaxel • AC → paclitaxel → trastuzumab	Sequential arm • HR 0.69, p < 0.001 • 71.8% versus 80.1% • 225 versus 165 events	Sequential arm • HR 0.88, p < 0.343 • 88.4% versus 89.3% • 108 versus 96 events	Perez *et al* (2011) [32]
NCCTG N9831 and NSABP B—31 trials (ǂ)	4046/8.4 years	• AC → paclitaxel • AC → paclitaxel + trastuzumab → trastuzumab	• HR 0.60, p < 0.001 • 62.3% versus 73.7% • 680 versus 473 events	• HR 0.63, p < 0.001 • 75.2% versus 84.0% • 418 versus 286	Perez *et al* (2014) [5]
HERA trial (§)	3401/ 4 years	• four cycles standard chemotherapy • four cycles standard chemotherapy → trastuzumab	• HR 0.76, p < 0.001 • 72.2% versus 78.6% • 458 versus 369 events	• HR 0.85, p < 0.11 • 87.7% versus. 89.3% • 213 versus 182 events	Gianni *et al* (2011) [50]
FNCLCC—PACS04 trial (§)	3010/3 years	• FEC or epirubicin plus docetaxel • FEC or epirubicin plus docetaxel → trastuzumab	• HR 0.86, p = 0.41 • 77.9% versus 80.9% • 70 versus 59 events	• HR 1.27, p = NR • 96% versus 95% • 18 versus 22 events	Spielmann *et al* (2009) [51]
FinHer trial (ǂ)	232/5.1 years	• Docetaxel or vinorelbine → FEC • Docetaxel or vinorelbine with trastuzumab → FEC	• HR 0.65, p = 0.12 • 73.3% versus 80.9% • 31 versus 22 events	• HR 0.55, p = 0.09 • 82.3% versus91.3% • 21 versus 12 events	Joensuu *et al* (2006) [6, 7]
**Meta-analysis**					
NA	11991*/3 years	NA	• HR 0.6, 95% CI 0.5–0.71	• HR 0.66, 95% CI 0.57–0.77	Moja *et al* (2012) [8]

ǂ Concurrent chemotherapy and trastuzumab.

§ Sequential chemotherapy and trastuzumab.

FEC–fluorouracil plus epirubicin plus cyclophosphamide.

AC–Doxorubicin plus cyclophosphamide.

NR–not reported. NA–not applicable.

* DFS and OS analysis included different amounts of patients.

**Table 2.** Summary of results from major trials addressing trastuzumab benefit in the neoadjuvant treatment of BC.

Study	Number of patients	Comparison treatment	pCR (breast and lymph nodes) with no anti-HER2 therapy, % (95% CI)	pCR (breast and lymph nodes) for T, % (95% CI)	pCR (breast and lymph nodes) for anti-HER2 comparator (if applic.), % (95% CI)	pCR (breast and lymph nodes) for T plus anti-HER2 comparator (if applic.), % (95% CI)	Reference
**Trastuzumab only**							
MD Anderson study	42	• P → FEC • P + T → FEC + T	26.3 (9–51)	65.2 (43–84)	NA	NA	Buzdar *et al* (2005) [16]
MD Anderson study (second cohort)	22	• P → FEC • P + T → FEC + T	NA	54.5 (32–75.6)	NA	NA	Buzdar *et al* (2007) [17]
NOHA study	235	• CT (including combinations of doxorubicin, paclitaxel, cyclophosphamide, methotrexate and 5—fluorouracil) • CT + T	19 (NR)	38 (NR)	NA	NA	Gianni *et al* (2010, 2013) [18, 19]
GeparQuattro	1509 of which 445 HER2 positive	• EC → D (+/—Cap) [administered in HER2 negative pts] • EC → D + T (+/— Cap) [administered in HER2 positive pts]	48.0 (NR)	62.5 (NR)	NA	NA	Untch *et al* (2010) [20]
**Trastuzumab versus or plus lapatinib**							
GeparQuinto	620	• EC + T → D + T • EC + L → D + L	NA	30.3 (25.2–35.8)	22.7 (18.2–27.8)	NA	Untch *et al* (2012) [21]
CHER-LOB	121	• P + T → FEC + T • P + L → FEC + L • P + T + L → FEC + T + L	NA	25 (13.1–36.9)	26.3 (14.5–38.1)	46.7 (34.4–58.9)	Guarneri *et al* (2012) [22]
NeoALTTO	455	• T → P + T • L → P + L • T + L → P + T + L	NA	27.6 (97.5% CI 20.5–36.2) ER negative group: 36.49 (25.60–48.49)	20.0 (97.5% CI 13.9–27.3) ER negative group: 33.78 (23.19–45.72)	46.8 (NR) ER negative group: 61.33 (49.38–72.36)	Baselga *et al* (2012) [23]
NSABP B41	529	• AC → P + T • AC → P + L • AC → P + T + L	NA	49.4 (41.8–56.5) ER negative group: 58.2 (44.1–69.9)	47.4 (39.8–54.6) ER negative group: 54.9 (42.6–65.6)	60.2 (52.5–67.1) ER negative group: 69.8 (56.8–79.5)	Robidoux *et al* (2013) [52]
CALGB 40601	305	• P + T • P + L • P + T + L (prematurely closed)	NA	Only breast pCR reported: 40 (32–49)	Only breast pCR reported: 32 (22–44)	Only breast pCR reported: 51 (42–60)	Carey *et al *(2013) [53]
TRIO US B07	130	• TC + T • TC + L • TC + T + L	NA	43 (NR) ER negative group: 58% (NR)	25 (NR) ER negative group: 42% (NR)	52 (NR) ER negative group: 68% (NR)	Hurvitz *et al* (2013) [54]
TBCRC 006	66	• T + L (Let +/- LHRHa if premenopausal)	NA	NA	NA	22 (NR) ER negative group: 28 (NR) ER positive group: 18 (NR)	Rimawi *et al* (2013) [55]
LPT 109096	100	FEC → P + T FEC → P + L FEC → P + T + L	NA	54 (NR)	45 (NR)	74 (NR)	Holmes *et al* (2011) [56]
**Trastuzumab versus or plus pertuzumab**							
NeoSphere	417	NA	• T + D • T + Pe + D • (- T + Pe) • Pe + D	29.0 (20.6–38.5) ER negative group: 36.8 (24.4–50.7)	24.0 (15.8–33.7) ER negative group: 30.0 (17.9–44.6)	45.8 (36.1–55.7) ER negative group: 63.2 (49.3–75.6)	Gianni *et al* (2012) [25]

AC–Doxorubicin plus cyclophosphamide;

Cap–Capecitabine;

C–Chemotherapy;

D–Docetaxel;

EC–Epirubicin plus cyclophosphamide;

FEC–fluorouracil plus epirubicin plus cyclophosphamide;

L–Lapatinib;

LHRHa–Luteinising Hormone Releasing Hormone agonist;

NA–not applicable;

NR–not reported;

P–Paclitaxel;

Pe–Pertuzumab;

Pts–Patients;

T–Trastuzumab

**Table 3.** Optimal treatment duration, results compared to one year standard therapy.

	Study	Design	NR patients/follow-up	DFS	OS	Reference
**Shorter duration**	PHARE	Non-inferiority (six months)	3380/2 years	• HR 1.28, p = 0.29 • 93.8% versus 91.1% • 175 versus 219 events	• HR 1.46* • 96.1% versus 94.5% • 66 versus 93 events	Pivot *et al* (2013) [26]
**Longer duration**	HERA	Superiority (two years)	3402/8 years	• HR 0.99, p = 0.86 • 76% versus 75.8% • 367 versus 367 events	• HR 1.05, p = 0.63 • 88.7% versus 86.9% • 274 versus 278 events	Goldhirsch *et al* (2013) [27]

* Proportional hazards could not be reasonably accepted for overall survival (lack of proportional hazards)

**Table 4.** Summary of pertuzumab and trastuzumab emtansine (T-DM1) (neo)adjuvant phase III studies.

Study	Treatment setting	Intervention	Comparator	Recruitment target and status
APHINITY (NCT01358877)	Adjuvant therapy	Chemotherapy + pertuzumab + trastuzumab	Chemotherapy + placebo + trastuzumab	4808; completed
KATHERINE (NCT01772472)	Adjuvant therapy post neoadjuvant therapy	TDM1	Trastuzumab	1484; ongoing
KAITLIN (NCT01966471)	Adjuvant therapy	TDM1 + pertuzumab	Trastuzumab + pertuzumab + taxane	2500; ongoing
